# Quantum Molecular Devices

**DOI:** 10.1021/acsphyschemau.3c00077

**Published:** 2024-02-26

**Authors:** Ronnie Kosloff

**Affiliations:** Institute of Chemistry, Hebrew University of Jerusalem, Jerusalem 9190401, Israel

**Keywords:** Quantum Thermodynamics, Ultracold Chemistry, Coherent Control, Molecular Electronics, Laser Cooling, Quantum Computing

## Abstract

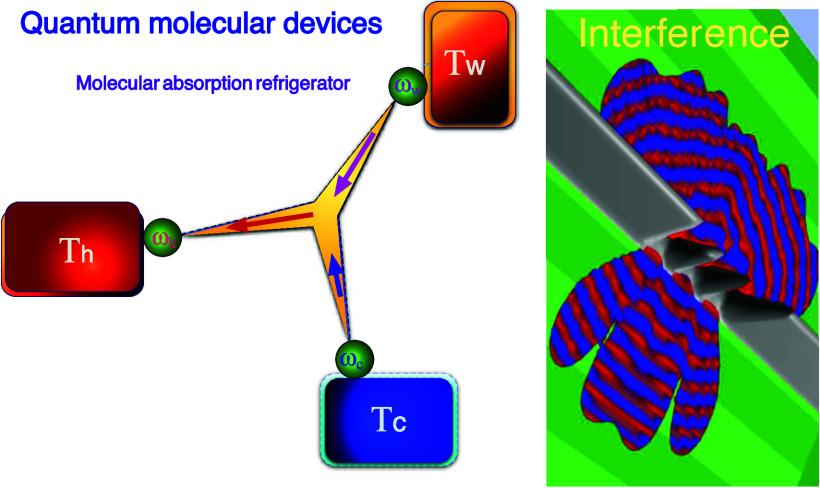

Miniaturization has been the driving force in contemporary technologies. However, two main obstacles limit further progress: additional reduction in size has reached its quantum limit, and lithography has reached its threshold. Future progress requires tackling three challenges: chemical synthesis of a complete device, active cooling for exploiting quantum characteristics, and quantum coherent control for operation. Chemical synthesis replaces the current top-bottom approach to manufacturing with bottom-up synthesis from elementary building blocks. New ultracold synthetic methods should be developed. An additional challenge is the active cooling of molecules, where the bottleneck is entropy removal. Notably, the current solution, namely, diffusion, is too slow. A coherent approach offers a possible solution; specifically, quantum coherent control is the method of choice for manipulating ultracold matter. Finally, the many degrees of freedom of molecules should be an asset that allows the design and implementation of complex tasks such as sensing communication and computing.

## Introduction

We are currently living in the information era. Accordingly, many aspects of our day-to-day life require retrieval of information or information processing. In this cybernetics world, the main assets are virtual. Nevertheless, in reality, information must become physical.

The history of information storage begins with cumbersome clay tablets containing several bits of information that became denser with time. The invention of printing is considered a milestone in this progress. It enhanced the distribution of knowledge but was also one of the first steps of information processing, i.e., transforming information from one media to another. Printing techniques, particularly lithography, are still at the heart of microelectronic data processing devices. Through the decades, information storage density increased exponentially, reaching ∼10^13^ bits/cm^2^. Other information processing devices follow this trend, adhering to Moore’s empirical law.^1^

Subsequently, has the existing strategy of top-down lithography reached its limits?^2^ The current basic technology is silicon semiconductor integrated circuits. Other physical techniques, such as gallium arsenide-based semiconductors with a faster clock speed, have been suggested. However, these alternatives to silicon have not lived up to their promise as general-purpose devices. A more revolutionary idea is molecular electronics,^3^ a bottom-up approach, assembling the device from elementary building blocks.^4−6^ However, this approach has not yet materialized into a working device. Concurrent with the increase in storage density, chip manufacturers’ engineering efforts have miniaturized the elementary device to almost the molecular level, rendering molecular electronics superfluous. Therefore, we anticipate that, to proceed beyond the molecular/atomic level, a new paradigm must be sought.

Quantum information processing has the promise of such a paradigm since the information density can be made exponentially larger through the superposition principle. Accordingly, the quantum supremacy experiment based on 53 qubits^7^ has potentially more storage capacity than all existing combined computer memory. This superconducting-based device was manufactured using lithographic techniques. However, the required technology is still in its infancy. Specifically, the main current obstacle is decoherence, which limits the number of consecutive operations to ∼12. Another astonishing observation is the discrepancy between the size of the quantum device ∼1 cm^2^ to the size of the dilution refrigerator ∼1 m^3^ required to maintain the device at superconducting temperature. This technological and scientific challenge points to the question: Can a hand-held quantum computer be realized?

At present, the anticipated vision is a molecular bottom-up approach assembling a quantum information processing device from elementary components. We now address the challenges facing such a concept.

The current working model of physical chemistry addresses the electrons as quantum components and the nuclei as semiclassical. This viewpoint is based on the ambient thermal energy scale, where electrons typically occupy the ground state within the Born–Oppenheimer approximation. High frequency vibrational degrees of freedom are populated primarily in the ground state. All degrees of freedom with energy below ∼200 cm^–1^ are thermally mixed, overshadowing any quantum phenomena. There are some exceptions for nuclear quantum effects; one can find evidence for quantum tunneling and zero point energy, such as in kinetic isotope effects.^8,9^ Nevertheless, for the majority of chemical phenomena, this framework is adequate.

A simple solution for enhancing the quantumness is to cool the molecular system so that all degrees of freedom become quantum. Therefore, if we can reach an operation temperature in the nano-Kelvin regime, practically all degrees of freedom will occupy the ground state.

Ultracold molecules are only a sufficient condition for constructing a quantum device. In addition, a control mechanism is required to fulfill the functions of writing information, information processing, and reading out. For this purpose, two basic models are considered:

- Classical control of a quantum device.
- Autonomous, fully quantum information processing.

External classical control relies on a time-dependent control Hamiltonian. By manipulating the control field, the dynamics can generate quantum gates.^10−12^ The model relies on an external pulse generator manipulating the quantum system. This is the current model of all attempts at realizing quantum computing. Typical working frequencies are slow compared to silicon semiconductors in the ∼10 GHz range.

The autonomous model is a fully quantum model, which, at this stage, is only in its infancy. Additionally, the model requires a fully quantum circuit with feedback.^13^ The current idea is to employ reinforcement learning to achieve the computation objective.^14^

## Cooling Molecules

A prerequisite for *quantumness* is cooling, which is a process of removing entropy. Ideally, one desires to reach as close as possible to the zero entropy ground state of the system. Any pure state with zero entropy is thermodynamically equivalent to an absolute zero temperature state. This equivalence stems from the fact that the von Neumann entropy is invariant to the unitary dynamics of isolated systems. This fact also means that cooling requires nonunitary evolution, which is possible only in an open quantum system. Therefore, the cooling setup employs an external entropy sink used to remove entropy.

Cooling processes are limited by the III-law of thermodynamics.^15^ In a modern context, the unattainability principle^16^ requires infinite resources to reach a pure state. Consequently, in the case of molecular cooling, the more we attempt to purify our system, the more resources must be invested in this task.^17,18^

**Figure 1:**
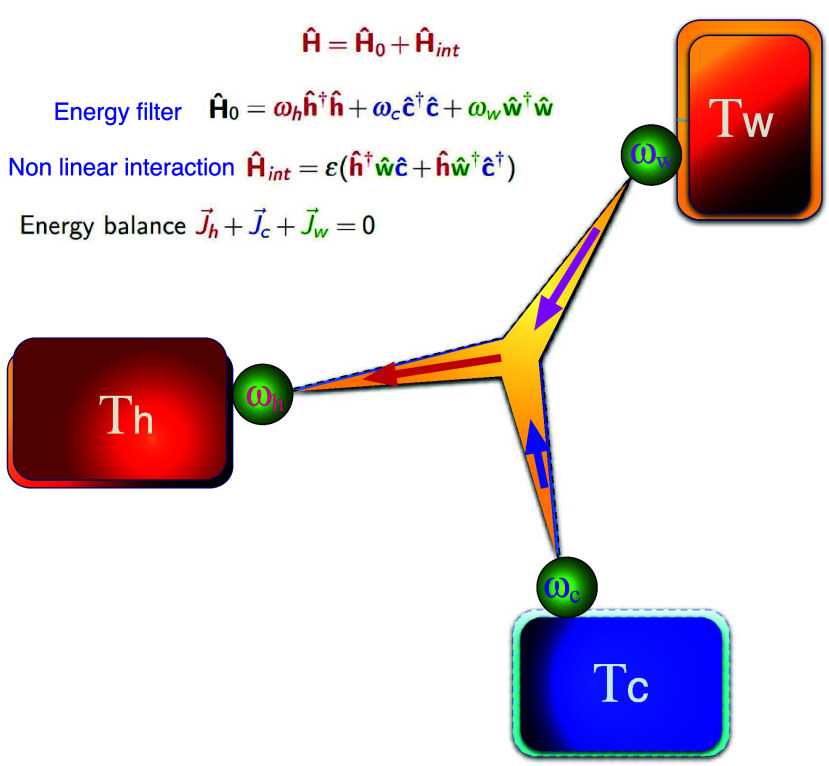
Tricycle: A universal quantum refrigerator. The system comprises three energy filters for a cold bath ω_c_, a hot bath ω_h_, and a power source bath ω_w_. A nonlinear interaction induces the heat current from the power source to the hot bath to pump heat from the cold bath. The optimal resonance condition is ω_w_ + ω_c_ = ω_h_.

The cooling process can be analyzed by employing the basic universal quantum refrigerator model (Figure 1).^19^ It operates by pumping heat from the cold bath and dumping it into the hot bath. This operation requires power obtained by either a driving field or a heat source hotter than the hot bath.^20^ Thermodynamically, the device transfers the downhill current from the power bath to the hot bath to extract heat from the cold bath, maintaining a positive global entropy production.

Entropy can be removed from a quantum system by two major means: diffusive or ballistic. The diffusive process removes entropy by independent particle collisions. Ballistic transport is a wave phenomenon propagating the excitation through the medium with the speed determined by the group velocity of the wave propagation. In macroscopic refrigerators, heat is removed almost exclusively by diffusive processes. As a result, refrigerators are large and cumbersome, with heat exchangers occupying large surface areas. An example of diffusive cooling at the molecular level is known as buffer gas cooling.^21,22^ Ultimately, diffusive heat removal for a quantum device is slow and very difficult to miniaturize.

The alternative, ballistic heat removal, can potentially miniaturize the device. The primary example is laser cooling. A heat engine analysis identifies the laser light as the power source, where the molecular device to be cooled acts as the cold bath, while the scattered light is equivalent to the entropy sink or the hot bath. The scattered light ballistically carries away the entropy. Other wave phenomena can play a similar role, for example, atomic Bose–Einstein Condensate (BEC) as a coolant,^23^ superconducting Cooper-pair electrons, or a BEC composed of polarons.^24^

### Difficulty in Cooling Molecules

The process of cooling molecules comes hand in hand with trapping. The first step in cooling molecules to ultracold temperatures is trapping them, which is an ongoing challenge. For molecular ions, electrodynamical traps have been constructed, such as the Paul trap^25^ or Zajfman trap.^26^ For neutral molecules, polarization of optical-based traps has been realized.^27^ For paramagnetic molecules, magnetic traps have been used.^28,29^ These traps can be categorized by depth, defined by the maximum temperature at which molecules can still be trapped. The challenge is to design a custom-made trap for a specific molecule.

Once trapped, all molecular degrees of freedom are localized and become discrete. Cooling a degree of freedom can be interpreted by a ground state occupation close to one. The molecular modes of motion span a huge range of frequencies, complicating the cooling process. The most difficult degrees of freedom to cool are the low-frequency ones, typically translation, rotation, fine structure, and hyperfine structure.

The generic method of cooling is optical pumping.^30^ In a nutshell, the scheme is based on selectively exciting the system to a high-frequency electronic transition. Spontaneous emission will repopulate the ground electronic states. To achieve cooling, at least one of the quantum states on the lower electronic manifold must be dark, meaning it is not excited by the laser radiation.^31^ After numerous cycles of excitation and spontaneous emission, the population will accumulate in the dark states, increasing the quantum purity of the molecule. Analyzing the cooling process from a thermodynamic perspective, the process can be classified as a power-driven refrigerator removing entropy from the lower electronic manifold and dumping it into the entropy sink of the scattered light.^32^

Current examples of optical pumping include cooling of NV centers in diamonds,^33^ sideband cooling of optomechanical devices,^34,35^ and cooling molecules trapped by optical tweezers.^36^ Moreover, miniaturization is allowed since the removal of entropy in optical pumping is ballistic.

The challenge is, therefore, to explore additional ballistic cooling mechanisms specifically tailored for molecules.

## Ultracold Chemistry

One of the pillars of chemistry is synthesis, the amazing ability to design and create a molecule by demand. This feature is based on controlling the kinetics and thermodynamics of the process in order to stabilize the products. In solution synthesis, for example, the reaction products are stabilized by entropy generation in the solvent, thus suppressing a back-reaction.

This synthetic ability should be harnessed to generate quantum molecular devices. Molecular electronics also share this vision. In addition, in order to construct quantum devices, the synthesized molecules should be cooled to operating temperatures, which can be difficult to reach. In addition, the coolant to be used should not react with the device. Currently, the popular choice is a mixture of ^3^He and ^4^He from a dilution refrigerator. To obtain a colder coolant, a Bose–Einstein Condensate (BEC) can be considered.^37,38^ The limitations stem from the fact that any reaction between the molecular device and the coolant will boil off the coolant. An example is molecules inside a He droplet, where the temperature is set by the evaporation energy.^39^ In a mixture of ^3^He and ^4^He, it is in the mK range.

An alternative approach to ultracold chemistry is synthesizing the molecules from components at ultracold temperatures. Chemistry in such temperatures requires a reconsideration of the main principles. In this case, chemical reactions have to be executed under constant cooling. A current example would be the chemical reactions of laser-cooled ion crystals in Paul traps,^40^ in which the constant cooling removes entropy and stabilizes the product.

A universal synthetic method of laser-cooled entities can be carried out by photoassociation.^41^ The basic scheme starts from two stable reactants that are constantly cooled. As a result, the reactants occupy the mutual ground electronic surface, typically with very weak interaction between them. If the reactants possess dipoles, an effective repulsion interaction can be engineered.^42^ Upon electronic excitation, the interaction between the reactants becomes attractive, causing molecular fission. A stabilization step is now required, either employing spontaneous emission, removing the electronic excitation, and transferring the entropy ballistically away or selectively dissociating the entity to the desired products.^43,44^

**Figure 2:**
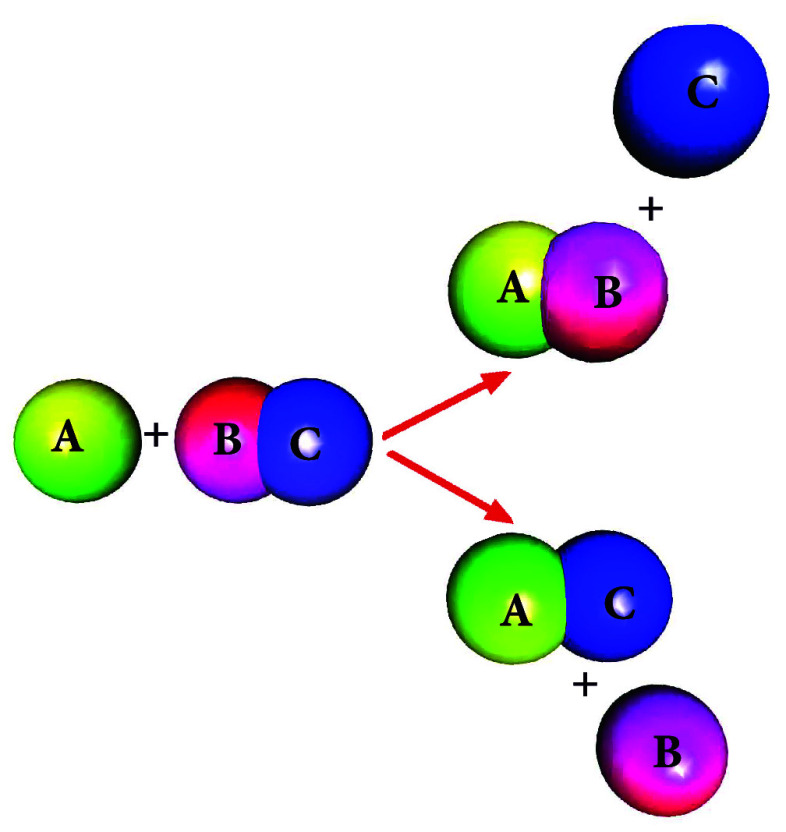
A + BC binary reaction: Control of the rearrangement channels. As an explicit example, we use Mg + LiH → MgLi + H → MgH + Li.

When the field of coherent control was conceived, the dream had been to control the synthesis of molecules,^45−51^ where the agent of control was quantum interference.^52^ The theoretical dream was realized in numerous experiments, of which the overwhelming majority concentrated on photodissociation. Only a handful of experiments addressed the binary chemical reaction.^53^ An outstanding experimental challenge is the control of the reaction of the type (cf. Figure 2).
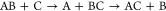
In this case, interference should enhance the desired product and suppress the alternative.

Quantum control of binary reactions requires pre-entangling the reactants.^54,55^ This issue is solved by photoassociation, which selects pre-entangled reactants. Selective dissociation can, in principle, lead to the desired ABC control. Experimentally, this task has not yet been achieved.

### Chemical Reactions in a Condensate

When the thermal de Broglie wavelength becomes comparable to the interparticle distance, the realm of ultracold chemistry emerges. This constitutes the transition from a particle description to a wave-like description.^56−58^ What are the consequences of this transition? Essentially, the individual molecular properties are replaced with collective phenomena.

In the condensate, the rate of the chemical reaction is modified. Bose stimulation can lead to a nearly complete selectivity of the collective N-body process, indicating a novel type of ultraselective quantum degenerate chemistry.^59,60^ Currently, experimental realizations are limited due to the few molecular BECs that have been produced. An interesting twist is a chemical reaction in a Polariton condensate.^61^

### Quantum Molecular Devices

The ultimate miniaturization challenge is to synthesize a complete quantum device from a single molecule. The realization of a molecular refrigerator is such an example. The idea is to synthesize the universal device shown in Figure 1. A hint of how such an endeavor can be achieved can be obtained from the molecular refrigerator realization employing three ions in a laser-cooled Paul trap.^62^ The three normal modes of the ions constitute the energy filters and obey the resonance condition ω_c_ + ω_w_ ≈ ω_h_. The nonlinear intermode coupling generates the three-body interaction . In addition, the molecule should connect by three leads to the heat baths. Another hint arises from the realization of the universal refrigerator in a Josephson junction superconduction-based device,^63^ where energy filters are realized by three superconducting qubits. The nonlinear interaction is realized by employing an additional level, qutrit, on one of the qubits and a four-wave mixing type of interaction.

The realization of an efficient molecular refrigerator able to cool to ultralow temperatures will have numerous applications. Such a device can lead to a hand-held quantum processor.

**Figure 3:**
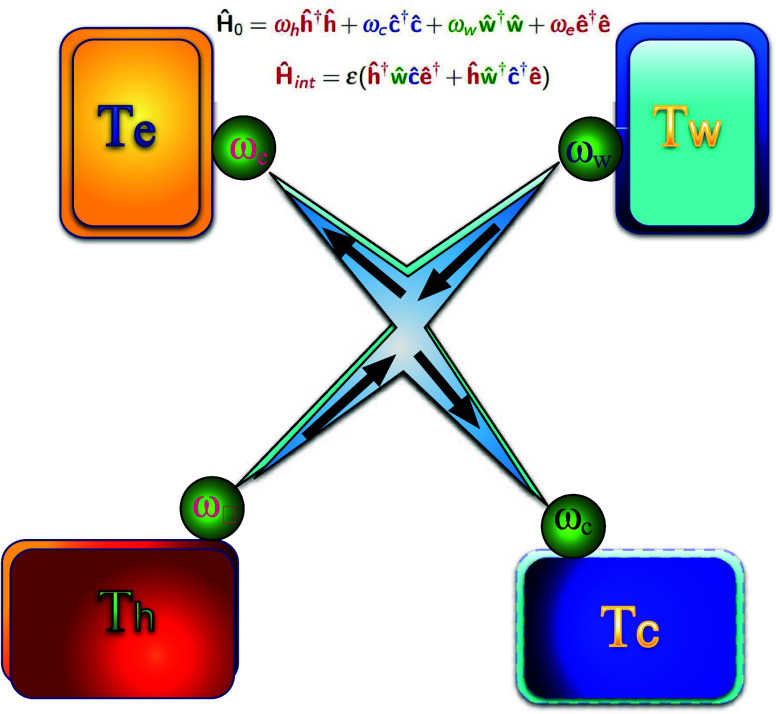
Four-terminal quantum pump. The device is composed of four energy filters for a cold bath ω_c_, a hot bath ω_h_, a power source bath ω_w_, and a sink ω_e_. A nonlinear interaction induces the heat current from the hot bath to the cold bath to pump heat from the work bath to the dump bath. Optimal resonance condition: ω_w_ – ω_e_ = ω_h_ – ω_c_.

Another challenging molecular device is a four-terminal heat or charge pump (Figure 3) in which counter heat flow from a hot to a cold bath pumps the charge against a potential bias or vice versa. The basic nonlinear interaction is a four-wave-mixing type. This operation requires connecting the device to four leads.

The final challenge is to synthesize an integrated device from the components. Such a device will contain an integrated refrigerator for internal cooling and ballistic cooling to carry away the entropy. Finally, it will contain a logical component able to perform quantum information processing.

## Summary

A quantum molecular device constitutes a bottom-up approach to miniaturizing quantum information processing. The device’s small size has the advantage of maintaining coherence for many consecutive operations. The challenges for reaching this goal are cooling, control, and synthesis. The theory accompanying these developments requires further progress in quantum dynamics and, in particular, quantum dynamics of open systems.

The route to realizing quantum molecular devices is modular and can be partitioned into elementary steps:

1. Selective cooling and trapping of molecules.
2. Novel synthetic methods appropriate for ultracold chemistry, including coherent control and chemical reactions in a BEC condensate.
3. Experimental realization of the A + BC reaction control.
4. Synthesis and operation of a molecular refrigerator.
5. Generating an integrated molecular device.

Quantum molecular devices are currently at our doorstep. Their operation principles of superposition, entanglement, and coherence are novel and, therefore, require a change of paradigm to a fully quantum framework for physical chemistry.

## References

[ref1] Moore G. E. Cramming more components onto integrated circuits, Reprinted from Electronics, volume 38, number 8, April 19, 1965, pp. 114 ff. IEEE solid-state circuits society newsletter 2006, 11, 33–35. 10.1109/N-SSC.2006.4785860.

[ref2] Shalf J. The future of computing beyond Moore’s Law. Philosophical Transactions of the Royal Society A 2020, 378, 20190061 10.1098/rsta.2019.0061. 31955683

[ref3] Ratner M. A brief history of molecular electronics. Nature Nanotechnol. 2013, 8, 378–381. 10.1038/nnano.2013.110. 23736207

[ref4] Mirkin C. A.; Ratner M. A. Molecular electronics. Annu. Rev. Phys. Chem. 1992, 43, 719–754. 10.1146/annurev.pc.43.100192.003443.

[ref5] Mathew P. T.; Fang F. Advances in molecular electronics: a brief review. Engineering 2018, 4, 760–771. 10.1016/j.eng.2018.11.001.

[ref6] Chen H.; Fraser Stoddart J. From molecular to supramolecular electronics. Nat. Rev. Mater. 2021, 6, 804–828. 10.1038/s41578-021-00302-2.

[ref7] Arute F.; Arya K.; Babbush R.; Bacon D.; Bardin J. C.; Barends R.; Biswas R.; Boixo S.; Brandao F. G.; Buell D. A.; et al. Quantum supremacy using a programmable superconducting processor. Nature 2019, 574, 505–510. 10.1038/s41586-019-1666-5. 31645734

[ref8] Karandashev K.; Xu Z.-H.; Meuwly M.; Vanicek J.; Richardson J. O. Kinetic isotope effects and how to describe them. Structural Dynamics 2017, 4, 061501 10.1063/1.4996339. 29282447 PMC5729036

[ref9] Romm L.; Citri O.; Kosloff R.; Asscher M. A remarkable heavy atom isotope effect in the dissociative chemisorption of nitrogen on Ru (001). J. Chem. Phys. 2000, 112, 8221–8224. 10.1063/1.481476.

[ref10] Palao J. P.; Kosloff R. Quantum computing by an optimal control algorithm for unitary transformations. Physical review letters 2002, 89, 188301 10.1103/PhysRevLett.89.188301. 12398642

[ref11] Glaser S. J.; Boscain U.; Calarco T.; Koch C. P.; Köckenberger W.; Kosloff R.; Kuprov I.; Luy B.; Schirmer S.; Schulte-Herbrüggen T.; et al. Training Schrödinger’s cat: Quantum optimal control: Strategic report on current status, visions and goals for research in Europe. European Physical Journal D 2015, 69, 1–24. 10.1140/epjd/e2015-60464-1.

[ref12] Koch C. P.; Boscain U.; Calarco T.; Dirr G.; Filipp S.; Glaser S. J.; Kosloff R.; Montangero S.; Schulte-Herbrüggen T.; Sugny D.; et al. Quantum optimal control in quantum technologies. Strategic report on current status, visions and goals for research in Europe. EPJ. Quantum Technology 2022, 9, 19 10.1140/epjqt/s40507-022-00138-x.

[ref13] Lipka-Bartosik P.; Perarnau-Llobet M.; Brunner N. Thermodynamic Computing via Autonomous Quantum Thermal Machines. arXiv, arXiv:2308.15905; 10.48550/arXiv.2308.15905,. PMC11758477 39231232

[ref14] Lamata L. Quantum reinforcement learning with quantum photonics; Photonics, 2021; p 33.

[ref15] Nernst W. Über die Beziehungen zwischen Wärmeentwicklung und maximaler Arbeit bei kondensierten Systemen. Ber. Kgl. Pr. Akad. Wiss 1906, 52, 933–940.

[ref16] Freitas N.; Gallego R.; Masanes L.; Paz J. P. Cooling to absolute zero: The unattainability principle. Thermodynamics in the Quantum Regime: Fundamental Aspects and New Directions 2018, 195, 597–622. 10.1007/978-3-319-99046-0_25.

[ref17] Levy A.; Alicki R.; Kosloff R. Quantum refrigerators and the third law of thermodynamics. Phys. Rev. E 2012, 85, 061126 10.1103/PhysRevE.85.061126. 23005070

[ref18] Taranto P.; Bakhshinezhad F.; Bluhm A.; Silva R.; Friis N.; Lock M. P.; Vitagliano G.; Binder F. C.; Debarba T.; Schwarzhans E.; et al. Landauer Versus Nernst: What is the True Cost of Cooling a Quantum System?. PRX Quantum 2023, 4, 010332 10.1103/PRXQuantum.4.010332.

[ref19] Kosloff R. Quantum thermodynamics: A dynamical viewpoint. Entropy 2013, 15, 2100–2128. 10.3390/e15062100.

[ref20] Cangemi L. M.; Bhadra C.; Levy A. Quantum Engines and Refrigerators. arXiv, arXiv:2302.00726; 10.48550/arXiv.2302.00726.

[ref21] Egorov D.; Lahaye T.; Schöllkopf W.; Friedrich B.; Doyle J. M. Buffer-gas cooling of atomic and molecular beams. Phys. Rev. A 2002, 66, 043401 10.1103/PhysRevA.66.043401.

[ref22] Gantner T.; Koller M.; Wu X.; Rempe G.; Zeppenfeld M. Buffer-gas cooling of molecules in the low-density regime: Comparison between simulation and experiment. Journal of Physics B: Atomic, Molecular and Optical Physics 2020, 53, 145302 10.1088/1361-6455/ab8b42.

[ref23] Hewitt T.; Bertheas T.; Jain M.; Nishida Y.; Barontini G. Controlling the interactions in a cold atom quantum impurity system. arXiv, arXiv:2310.02771; 10.48550/arXiv.2310.02771.

[ref24] Duda M.; Chen X.-Y.; Schindewolf A.; Bause R.; von Milczewski J.; Schmidt R.; Bloch I.; Luo X.-Y. Transition from a polaronic condensate to a degenerate Fermi gas of heteronuclear molecules. Nat. Phys. 2023, 19, 720–725. 10.1038/s41567-023-01948-1.

[ref25] Drewsen M.; Brøner A. Harmonic linear Paul trap: Stability diagram and effective potentials. Phys. Rev. A 2000, 62, 045401 10.1103/PhysRevA.62.045401.

[ref26] Dahan M.; Fishman R.; Heber O.; Rappaport M.; Altstein N.; Zajfman D.; Van Der Zande W. A new type of electrostatic ion trap for storage of fast ion beams. Review of Scientific instruments 1998, 69, 76–83. 10.1063/1.1148481.

[ref27] Anderegg L.; Cheuk L. W.; Bao Y.; Burchesky S.; Ketterle W.; Ni K.-K.; Doyle J. M. An optical tweezer array of ultracold molecules. Science 2019, 365, 1156–1158. 10.1126/science.aax1265. 31515390

[ref28] Segev Y.; Pitzer M.; Karpov M.; Akerman N.; Narevicius J.; Narevicius E. Collisions between cold molecules in a superconducting magnetic trap. Nature 2019, 572, 189–193. 10.1038/s41586-019-1446-2. 31391561

[ref29] Park J. J.; Lu Y.-K.; Jamison A. O.; Ketterle W. Magnetic trapping of ultracold molecules at high density. Nat. Phys. 2023, 19, 1567–1572. 10.1038/s41567-023-02141-0.

[ref30] Cohen-Tannoudji C.; Kastler A. Progress in optics; Elsevier, 1966; Vol. 5; pp 1–81.

[ref31] Bartana A.; Kosloff R.; Tannor D. J. Laser cooling of molecules by dynamically trapped states. Chem. Phys. 2001, 267, 195–207. 10.1016/S0301-0104(01)00266-X.

[ref32] Kosloff R.; Levy A. Quantum heat engines and refrigerators: Continuous devices. Annu. Rev. Phys. Chem. 2014, 65, 365–393. 10.1146/annurev-physchem-040513-103724. 24689798

[ref33] Kern M.; Jeske J.; Lau D.; Greentree A.; Jelezko F.; Twamley J. Optical cryocooling of diamond. Phys. Rev. B 2017, 95, 235306 10.1103/PhysRevB.95.235306.

[ref34] Riviere R.; Deleglise S.; Weis S.; Gavartin E.; Arcizet O.; Schliesser A.; Kippenberg T. J. Optomechanical sideband cooling of a micromechanical oscillator close to the quantum ground state. Phys. Rev. A 2011, 83, 063835 10.1103/PhysRevA.83.063835.

[ref35] Brubaker B. M.; Kindem J. M.; Urmey M. D.; Mittal S.; Delaney R. D.; Burns P. S.; Vissers M. R.; Lehnert K. W.; Regal C. A. Optomechanical ground-state cooling in a continuous and efficient electro-optic transducer. Physical Review X 2022, 12, 021062 10.1103/PhysRevX.12.021062.

[ref36] Mitra D.; Vilas N. B.; Hallas C.; Anderegg L.; Augenbraun B. L.; Baum L.; Miller C.; Raval S.; Doyle J. M. Direct laser cooling of a symmetric top molecule. Science 2020, 369, 1366–1369. 10.1126/science.abc5357. 32913101

[ref37] Ikemachi T.; Ito A.; Aratake Y.; Chen Y.; Koashi M.; Kuwata-Gonokami M.; Horikoshi M. All-optical production of dual Bose–Einstein condensates of paired fermions and bosons with 6Li and 7Li. Journal of Physics B: Atomic, Molecular and Optical Physics 2017, 50, 01LT01 10.1088/1361-6455/50/1/01LT01.

[ref38] Warner C.; Lam A. Z.; Bigagli N.; Liu H. C.; Stevenson I.; Will S. Overlapping Bose–Einstein condensates of Na 23 and Cs 133. Phys. Rev. A 2021, 104, 033302 10.1103/PhysRevA.104.033302.

[ref39] Huan C.; Kim S.; Yin L.; Xia J.; Candela D.; Sullivan N. NMR Studies of 3 He Droplets in Dilute 3 He-4 He Solid Solutions. Journal of Low Temperature Physics 2011, 162, 167–173. 10.1007/s10909-010-0260-x.

[ref40] Mølhave K.; Drewsen M. Formation of translationally cold MgH+ and MgD+ molecules in an ion trap. Phys. Rev. A 2000, 62, 011401 10.1103/PhysRevA.62.011401.

[ref41] Koch C. P.; Shapiro M. Coherent control of ultracold photoassociation. Chem. Rev. 2012, 112, 4928–4948. 10.1021/cr2003882. 22489790

[ref42] Guo M.; Ye X.; He J.; González-Martínez M. L.; Vexiau R.; Quéméner G.; Wang D. Dipolar collisions of ultracold ground-state bosonic molecules. Physical Review X 2018, 8, 041044 10.1103/PhysRevX.8.041044.

[ref43] Stwalley W. C.; Wang H. Photoassociation of ultracold atoms: a new spectroscopic technique. Journal of molecular spectroscopy 1999, 195, 194–228. 10.1006/jmsp.1999.7838. 10329265

[ref44] Gacesa M.; Byrd J. N.; Smucker J.; Montgomery J. A.; Côté R. Photoassociation of ultracold long-range polyatomic molecules. Phys. Rev. Res. 2021, 3, 023163 10.1103/PhysRevResearch.3.023163.

[ref45] Tannor D. J.; Rice S. A. Control of selectivity of chemical reaction via control of wave packet evolution. J. Chem. Phys. 1985, 83, 5013–5018. 10.1063/1.449767.

[ref46] Brumer P.; Shapiro M. Control of unimolecular reactions using coherent light. Chemical physics letters 1986, 126, 541–546. 10.1016/S0009-2614(86)80171-3.

[ref47] Tannor D. J.; Kosloff R.; Rice S. A. Coherent pulse sequence induced control of selectivity of reactions: Exact quantum mechanical calculations. J. Chem. Phys. 1986, 85, 5805–5820. 10.1063/1.451542.

[ref48] Tannor D. J.; Rice S. A. Coherent pulse sequence control of product formation in chemical reactions. Advances in Chemical Physics: Evolution of Size Effects in Chemical Dynamics Part 1 1988, 70, 441–523. 10.1002/9780470141199.ch10.

[ref49] Krause J. L.; Shapiro M.; Brumer P. Coherent control of bimolecular chemical reactions. J. Chem. Phys. 1990, 92, 1126–1131. 10.1063/1.458174.

[ref50] Rice S. A. New ideas for guiding the evolution of a quantum system. Science 1992, 258, 412–413. 10.1126/science.258.5081.412. 17833135

[ref51] Warren W. S.; Rabitz H.; Dahleh M. Coherent control of quantum dynamics: the dream is alive. Science 1993, 259, 1581–1589. 10.1126/science.259.5101.1581. 17733021

[ref52] Gordon R. J.; Rice S. A. Active control of the dynamics of atoms and molecules. Annu. Rev. Phys. Chem. 1997, 48, 601–641. 10.1146/annurev.physchem.48.1.601. 15012451

[ref53] Rybak L.; Amaran S.; Levin L.; Tomza M.; Moszynski R.; Kosloff R.; Koch C. P.; Amitay Z. Generating molecular rovibrational coherence by two-photon femtosecond photoassociation of thermally hot atoms. Phys. Rev. Lett. 2011, 107, 273001 10.1103/PhysRevLett.107.273001. 22243308

[ref54] Levin L.; Skomorowski W.; Rybak L.; Kosloff R.; Koch C. P.; Amitay Z. Coherent control of bond making. Physical review letters 2015, 114, 233003 10.1103/PhysRevLett.114.233003. 26196798

[ref55] Devolder A.; Brumer P.; Tscherbul T. V. Robust coherent control of two-body collisions beyond the ultracold regime. Physical Review Research 2023, 5, L042025 10.1103/PhysRevResearch.5.L042025.

[ref56] Carr L. D.; DeMille D.; Krems R. V.; Ye J. Cold and ultracold molecules: science, technology and applications. New J. Phys. 2009, 11, 055049 10.1088/1367-2630/11/5/055049.

[ref57] Jin D. S.; Ye J. Introduction to ultracold molecules: new frontiers in quantum and chemical physics. Chem. Rev. 2012, 112, 4801–4802. 10.1021/cr300342x. 22967213

[ref58] Quemener G.; Julienne P. S. Ultracold molecules under control. Chem. Rev. 2012, 112, 4949–5011. 10.1021/cr300092g. 22921011

[ref59] Moore M.; Vardi A. Bose-enhanced chemistry: Amplification of selectivity in the dissociation of molecular Bose–Einstein condensates. Physical review letters 2002, 88, 160402 10.1103/PhysRevLett.88.160402. 11955220

[ref60] Zhang Z.; Nagata S.; Yao K.-X.; Chin C. Many-body chemical reactions in a quantum degenerate gas. Nat. Phys. 2023, 19, 1466–1470. 10.1038/s41567-023-02139-8.

[ref61] Pannir-Sivajothi S.; Campos-Gonzalez-Angulo J. A.; Martínez-Martínez L. A.; Sinha S.; Yuen-Zhou J. Driving chemical reactions with polariton condensates. Nat. Commun. 2022, 13, 1645 10.1038/s41467-022-29290-9. 35347131 PMC8960839

[ref62] Maslennikov G.; Ding S.; Hablützel R.; Gan J.; Roulet A.; Nimmrichter S.; Dai J.; Scarani V.; Matsukevich D. Quantum absorption refrigerator with trapped ions. Nat. Commun. 2019, 10, 1–8. 10.1038/s41467-018-08090-0. 30643131 PMC6331551

[ref63] Aamir M. A.; Suria P. J.; Guzmán J. A. M.; Castillo-Moreno C.; Epstein J. M.; Halpern N. Y.; Gasparinetti S. Thermally driven quantum refrigerator autonomously resets superconducting qubit. arXiv arXiv:2305.16710; 10.48550/arXiv.2305.16710.

